# Gastric Ulcer from the Pressure of a Gastrostomy Tube: A Rare Cause of Upper Gastrointestinal Bleeding

**DOI:** 10.7759/cureus.2783

**Published:** 2018-06-11

**Authors:** Jamil M Shah, Abul B Shahidullah

**Affiliations:** 1 Department of Internal Medicine, The Brooklyn Hospital Center, Academic Affiliate of the Icahn School of Medicine at Mount Sinai, Clinical Affiliate of the Mount Sinai Hospital, New York, USA; 2 Department of Medicine, Henry J. Carter Specialty Hospital and Nursing Facility, New York, USA

**Keywords:** gastrostomy, percutaneous endoscopic gastrostomy, peg, complications of peg

## Abstract

Gastrostomy tube placement is a well-known procedure for obtaining permanent enteral access and providing long-term nutritional support. Although it is usually well tolerated, a diverse array of complications can occur. A rare, and often unrecognized, complication of gastrostomy tube placement is upper gastrointestinal bleeding secondary to a gastric ulcer caused by pressure from a gastrostomy tube bumper or balloon. Here, we present a case of an elderly woman who experienced hematemesis and bleeding around the gastrostomy site. This report should alert healthcare staff that excessive tightening of the gastrostomy tube retainer or prolonged traction of the gastrostomy tube can cause pressure necrosis manifesting as gastric ulceration.

## Introduction

In the United States, percutaneous endoscopic gastrostomy (PEG) tube placement is among the most frequently performed endoscopic procedures, with more than 200,000 performed annually, and many more are performed surgically with interventional radiology [1]. In fact, the procedure is now commonly performed across the world. Gastrostomy tubes are placed to provide enteral feeding to patients with disorders that make it difficult to swallow as well as to provide long-term nutritional support to patients with an inadequate oral intake. They are relatively safe and effective means by which to administer nourishment, hydration, and medications directly into the gastric lumen.

## Case presentation

**Figure 1:**
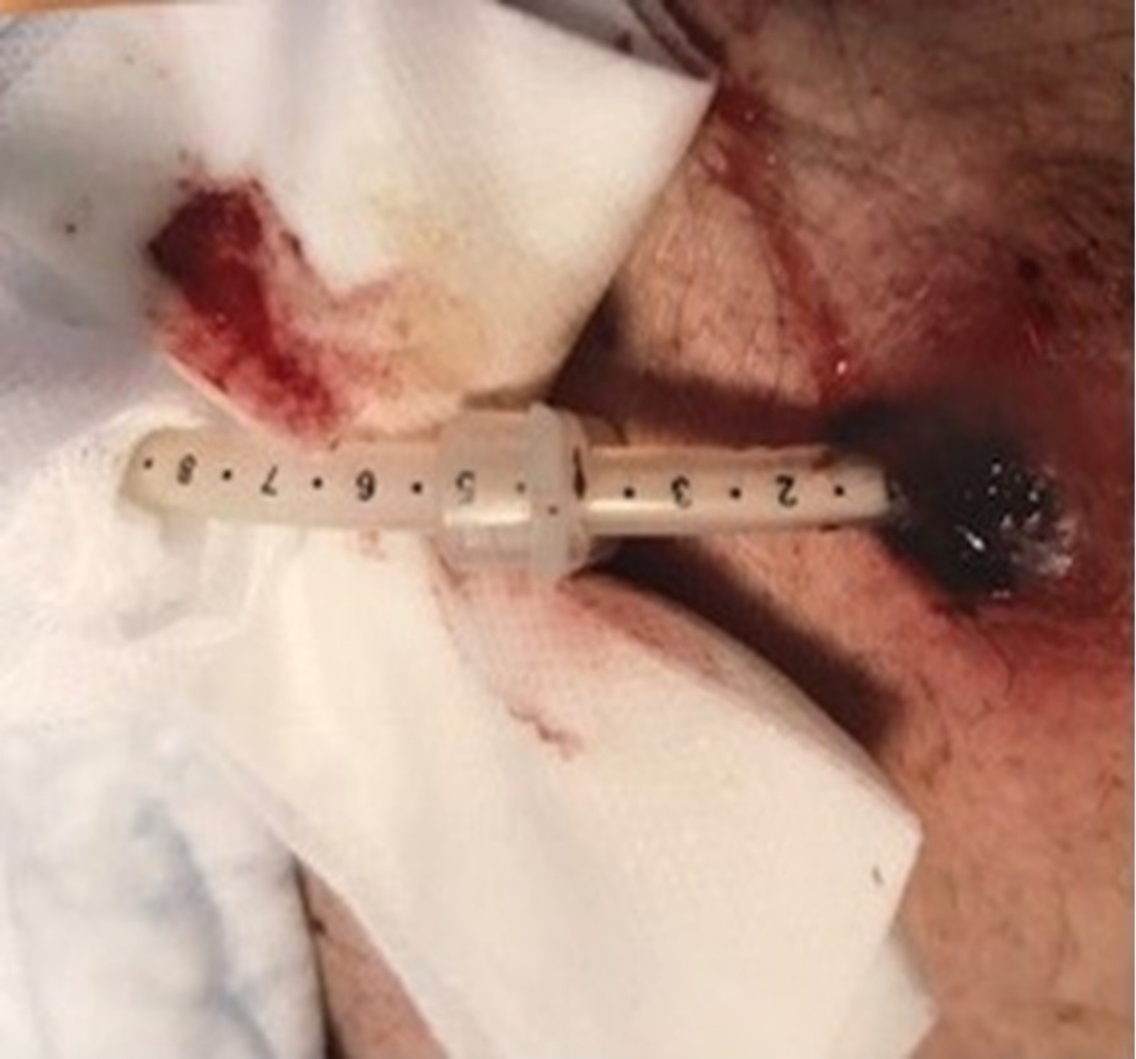
The patient’s abdomen with a gastrostomy tube Dried blood is visible at and around the gastrostomy site. The gastrostomy tube scale indicates that it is too far outside the abdomen.

An 80-year-old man residing in a nursing home, with a past medical history of a prior stroke, hypertension, hyperlipidemia, diabetes mellitus, dementia, and gastrostomy tube placement (inserted endoscopically two years before) due to dysphagia secondary to the stroke, presented to the emergency department (ED) with hematemesis and bleeding around the gastrostomy site. A review of his medication list revealed that he was taking aspirin 81 mg daily but no additional, nonsteroidal anti-inflammatory drugs (NSAIDs), antiplatelet drugs, or anticoagulants. On physical exam, he appeared exhausted. He was tachycardic (pulse rate 116 BPM), hypotensive (blood pressure 98/65 mm Hg), and febrile (temperature 100.6°F). The abdomen was soft and not distended, with no tenderness and normoactive bowel sounds. There was no guarding or rigidity. A gastrostomy tube was seen, and the scale indicated that the internal bumper had dislodged (Figure 1). Dried blood was seen at and around the gastrostomy site. On rectal exam, melena was discovered.

Aggressive intravenous hydration with normal saline was given. Laboratory testing was performed and the complete blood count (CBC) showed hemoglobin of 6.8 g/dL (12.0 - 15.5 g/dL) with an unknown baseline, hematocrit of 21% (34 - 48%), white blood cell count of 12.4 × 109/L (4.0 - 11.0 × 109/L), and platelet count of 382 × 109/L (150 - 450 × 109/L). The comprehensive metabolic panel (CMP) was remarkable for an elevated blood urea nitrogen of 42 mg/dL (7 - 22 mg/dL) and creatinine of 1.2 mg/dL (0.6 - 1.3 mg/dL). The liver function tests were normal. Two units of packed red blood cells were transfused.

**Figure 2:**
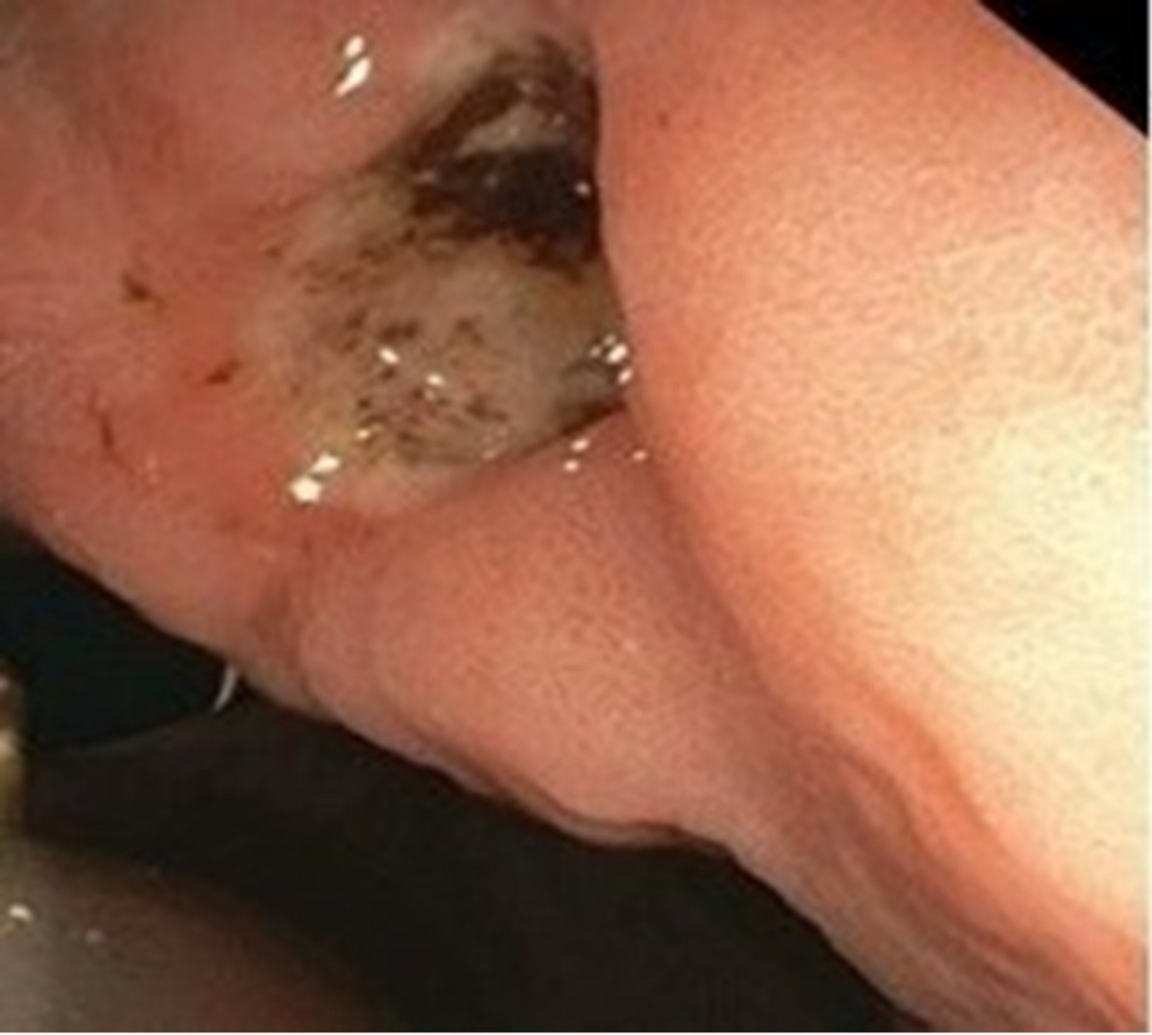
Endoscopic image of an ulcer in the gastric wall at the gastrostomy site A large blood clot is visible. There is no active bleeding.

Repeat laboratory testing performed the following day showed a hemoglobin of 9.0 g/dL (12.0 - 15.5 g/dL) and a hematocrit of 27% (34%-48%). Esophagogastroduodenoscopy (EGD) was performed and revealed a large gastric ulcer, with signs of recent bleeding, at the site of contact with the internal gastrostomy tube bumper, which had compressed and ulcerated the gastric wall (Figure 2). The dislodged gastrostomy tube was removed, and a new balloon gastrostomy tube was inserted, via the gastrostomy, into the stomach. The position of the balloon in the stomach was confirmed by endoscopy. The internal balloon was inflated with 15 mL of water.

Enteral feeding was successfully resumed the following day. Over the next few days, the patient’s clinical condition improved, and he returned to his nursing home.

## Discussion

**Figure 3:**
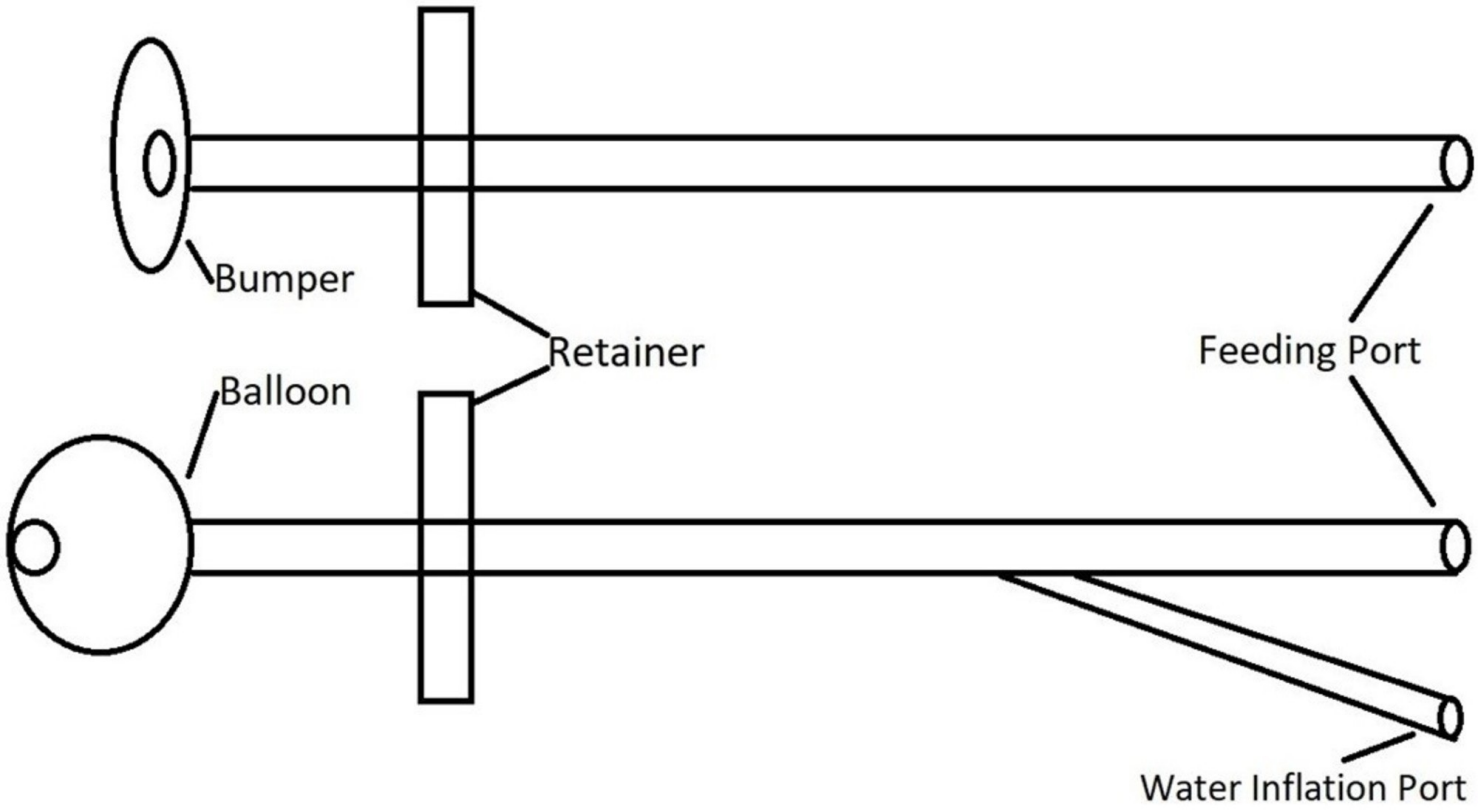
Components of bumper and balloon gastrostomy tubes

Gastrostomy tubes can be inserted via endoscopic, radiologic, and surgical methods. During placement by either method, a gastrocutaneous tunnel is created and a gastrostomy tube with either an internal bumper or an internal retention balloon is inserted (Figure 3). The bumper or balloon is located inside the stomach to prevent the spontaneous expulsion of the gastrostomy tube. The external retainer keeps the bumper or balloon in close contact with the inside wall of the stomach. It also helps to keep the abdominal and stomach walls in apposition for the future formation of a mature track. With the radiologic and surgical methods, sutures are placed to keep the abdominal and stomach walls in apposition. After six to eight weeks from the time of insertion, the gastrostomy track or gastrocutaneous fistula matures and the abdominal and stomach walls adhere permanently.

Although it is a relatively safe procedure, gastrostomy tube placement is associated with a diverse array of potential complications. In fact, despite the high success rate (>95%), major complications – those serious enough to require further intervention – have been reported in 0.4% to 4.4% of cases [2]. Some may occur immediately after gastrostomy tube placement and include pneumoperitoneum, esophageal, and gastric perforation, ileus, and injury to adjacent intra-abdominal organs, such as the liver and the colon [3]. Other potential complications tend to occur later on, after the gastrostomy tract has matured and include the deterioration of the gastrostomy site, buried bumper syndrome, and the formation of a colocutaneous fistula. Still other potential complications may occur at any time after gastrostomy tube placement and include peristomal leakage, infection, ulceration, bleeding, inadvertent removal of the tube, and gastric outlet obstruction [4-5]. One rare, and often unrecognized, complication of gastrostomy tube placement is upper gastrointestinal bleeding secondary to a gastric ulcer caused by pressure from a gastrostomy tube bumper or balloon, which can occur at any time following gastrostomy tube placement. The pathophysiology of such an ulcer is likely mucosal ischemia and pressure necrosis from an excessively tense application of the external retainer or the prolonged traction of the gastrostomy tube [6].

**Figure 4:**
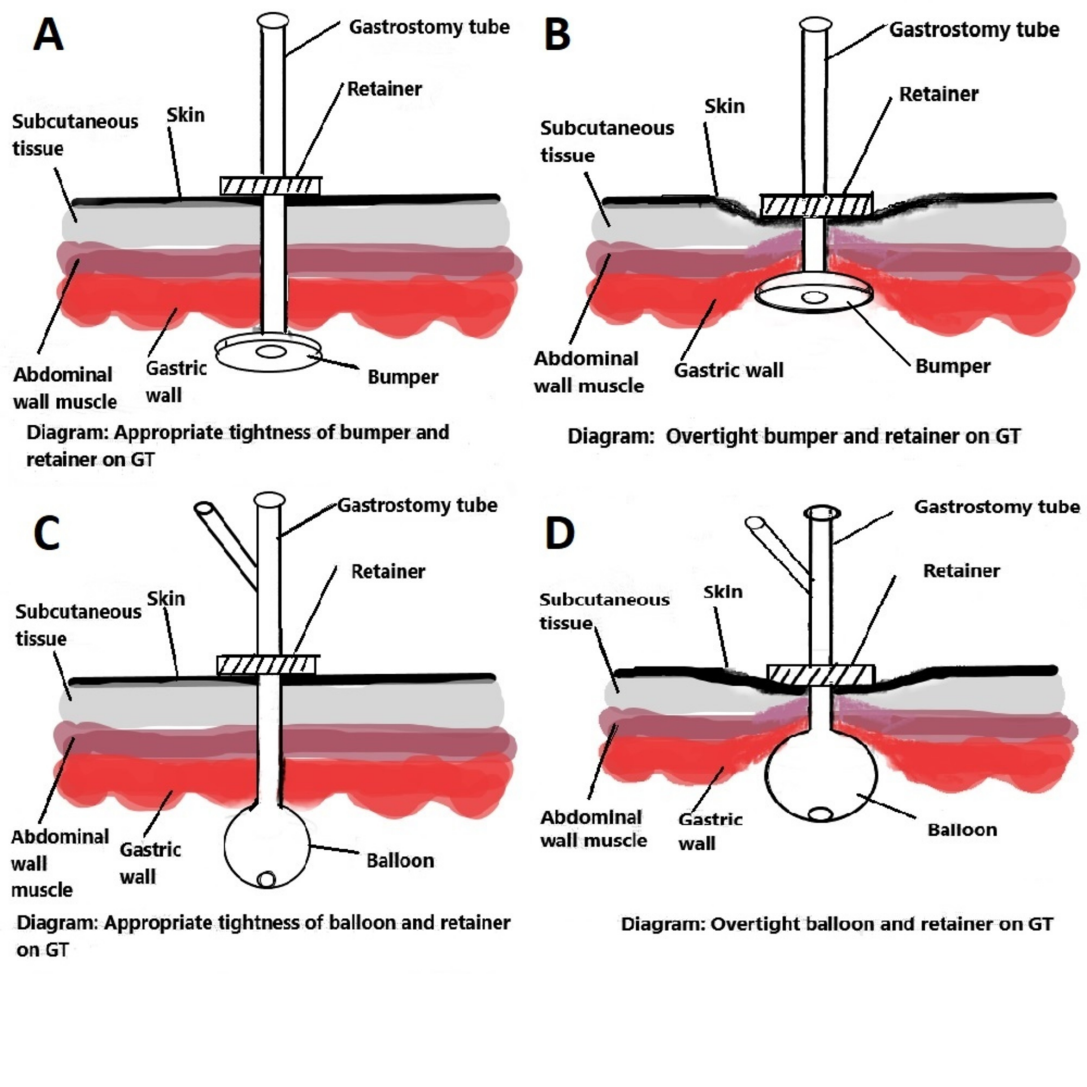
(A, B, C, D; left to right). Illustrations depicting the gastrostomy tube bumper or balloon on the inner side of the gastrostomy track as well as the retainer on the external side In between is the soft tissue of the stomach and abdominal walls. If the internal bump or balloon is pulled tightly against the gastric wall, the soft tissue becomes compressed. With this pressure, the gastric mucosa becomes ischemic, causing necrosis and the formation of an ulcer at the gastrostomy site.

Therefore, patients can develop pressure ulceration beneath the internal bumper or balloon and in the gastric wall related to the gastrostomy tube (Figure 4). Appropriate tension between the internal bumper or balloon and the external retainer helps to lessen this complication. While this phenomenon is usually observed in those with longstanding gastrostomy tubes, it can also be observed in those with gastrostomy tubes that have been recently placed, especially if the retainer is positioned such that the bumper or balloon is pulled tightly against the gastric wall. The ulcer often heals with the relaxing of the retainer, which permits the bumper or balloon to be freed from the gastric mucosa [6-8]. Clinicians should be aware of this phenomenon to ensure the proper functioning of the gastrostomy tube, avoid complications, and avoid the need for repeat endoscopy.

## Conclusions

In summary, gastrostomy tube placement is a relatively safe procedure and major morbidity is rare. Nevertheless, although gastrostomy care appears simple, it requires experience to avoid complications. Moreover, the number of patients who depend on gastrostomy tube feedings is growing throughout the world and complications are becoming ever more frequent. In clinical practice, physicians should be aware of and suspect gastric ulcer as a major complication of gastrostomy tube placement in a patient with upper gastrointestinal bleeding. This report can be used to emphasize the importance of following the gastrostomy tube manufacturers’ recommendations and exercising proper technique and caution.
